# Beyond Virtue-Signaling: Advancing Equity Through Design Justice and Public Health Critical Race Praxis

**DOI:** 10.1089/heq.2021.0075

**Published:** 2022-01-17

**Authors:** Hanna E. Huffstetler, Sarah E. Boland, Caitlin R. Williams, Dana K. Rice, Rohit Ramaswamy

**Affiliations:** ^1^ Health Behavior, Gillings School of Global Public Health, Chapel Hill, North Carolina, USA.; ^2^ Community and Behavioral Health, Colorado School of Public Health, Aurora, Colorado, USA.; ^3^ Maternal and Child Health and Gillings School of Global Public Health, Chapel Hill, North Carolina, USA.; ^4^ Public Health Leadership, Gillings School of Global Public Health, Chapel Hill, North Carolina, USA.; ^5^ Cincinnati Children's Medical Center, Cincinnati, OH, USA.

**Keywords:** design justice, critical race theory, public health critical race praxis, COVID-19

## Abstract

As public health mourns the inequitable loss of lives to coronavirus disease 2019 (COVID-19) and confronts other major social crises, practitioners must explicitly address systems of oppression in their everyday praxis. We describe how the principles of public health critical race praxis (PHCRP) and design justice (DJ) can advance equity in public health. We begin with an overview of PHCRP and DJ, and develop an integrated approach to facilitate community-led change. We apply this approach to the example of COVID-19 vaccine distribution and conclude with a call to action, arguing for PHCRP and DJ to become integral part of public health practice.

## Introduction

Globally, over 253 million people have contracted coronavirus disease 2019 (COVID-19) and more than 5.1 million have died, with mortality concentrated among vulnerable and oppressed populations.^1,2^ The life-saving promise of vaccines continues to be marred locally and globally by inequitable rollout. In the first six months of vaccine rollout, White people in the United States were significantly more likely than Black and Hispanic people to be vaccinated against COVID-19, despite increased risk among the latter groups for contracting and dying from the disease (though these disparities are beginning to narrow in many U.S. states).^3^

Such disparate health outcomes are the proverbial tip of the iceberg.^4^ Undergirding these disparities are flaws in the design and implementation of processes for developing and operationalizing public health responses, which are themselves the product of systemically biased values, assumptions, and beliefs. Such shortcomings are fueled by the interlocking forces of White supremacy, cisheteropatriarchy, capitalism, and settler colonialism that form the “matrix of domination.”^5^ This paradigm is closely related to the theory of “intersectionality,” which refers to how interconnected systems of race, class, and gender produce heightened exposure to oppression in ways that are not simply additive.^6^

As public health practitioners, we must understand how these frameworks illuminate deeply embedded power differences in our global history and culture that explicitly and implicitly influence the development of and decision-making around public health policies, programs, and practices.^7^ Cultivating such understanding is pressing in the current moment, as public health continues to grapple with historic harms to oppressed groups while also working to develop more just solutions to emerging and evolving health challenges.

Examining COVID-19 vaccination rollout using these frameworks illustrates how even well-intentioned efforts can reinforce oppression. For example, at a “walk-up equity clinic” to provide vaccinations to Black and Latinx residents in Massachusetts, people began queuing at 2:30 AM in front of a clinic that opened at 9:00 AM, forcing many to wait for hours.^8^ In contrast, at the primary by-appointment clinic, which was inaccessible through public transit, tens of thousands of higher-income residents breezed through their vaccinations.^9^

Thus, the default design for vaccine access—targeted toward White upper-class users—provided faster and more seamless care than the one intentionally designed to improve equity. Although no one intended to create *de facto* separate and unequal services, deeply rooted unconscious beliefs contributed to a design that reinscribed inequity.^10^

To develop solutions that advance rather than inadvertently impede equity, public health practitioners must be cognizant of systems of oppression and how they shape our lived realities, and then move beyond awareness to explicitly address those systems through their praxis. Merely acknowledging intersectional power asymmetries without taking concrete action results in the flattening and commodification of feminist thinking (e.g., intersectionality) and related movements—described by bell hooks as “partak[ing] of the ‘good’ that these movements produce without any commitment to transformative politics and practice.”^11,12^

In this article, we draw on two approaches that can facilitate intentional action: public health critical race praxis (PHCRP) and design justice (DJ). PHCRP is an antiracist approach to public health research, whereas DJ is an approach for empowering communities to lead design and social transformation. In shifting the locus of power away from institutions and toward community members, both frameworks provide tools for the development of effective community-centered solutions to otherwise intractable public health challenges. We explore how these approaches can be used together in a practical way to advance equity in public health policies, programs, and practices, using COVID-19 vaccination as an illustrative example.

## PHCRP and DJ: An Overview

**FIG. 1.**
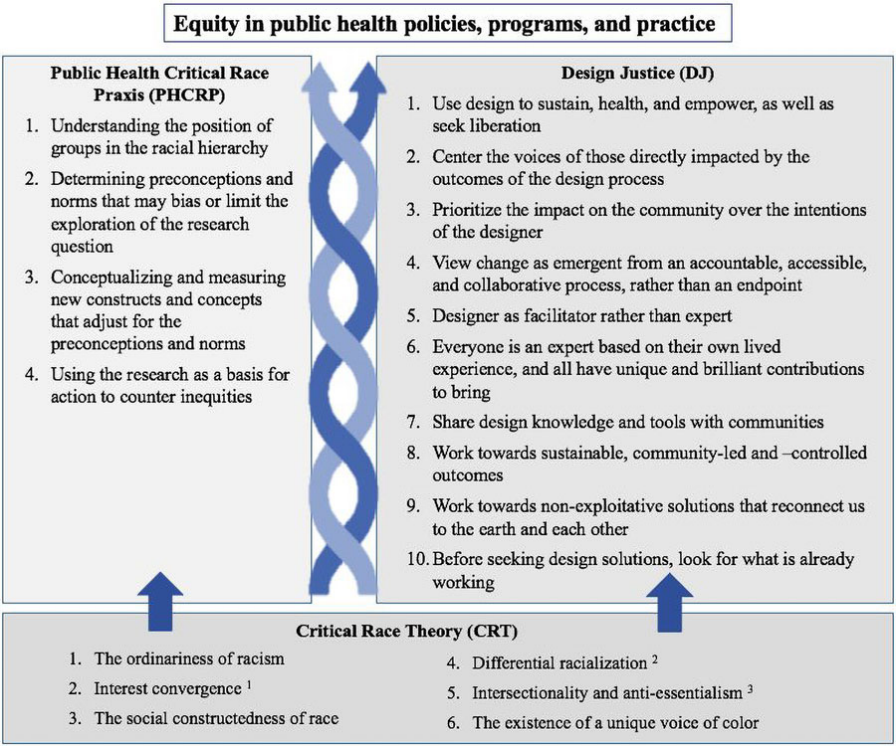
Critical race theory as a foundation for complementary praxes—Public Health Critical Race Praxis and Design Justice. ^1^Since racism works to the benefit of White communities, “interest convergence” posits that advancement for Black, Indigenous, and People of Color (BIPOC) communities only occurs when there is alignment between their interests and those of their White counterparts. ^2^“Differential racialization” recognizes how White society racializes BIPOC groups in different ways over time to accommodate White society's evolving needs and purposes. ^3^The concepts of “anti-essentialism” and “intersectionality,” respectively, refute the idea that individuals within a given social group share specific characteristics essential to the group, and describe how the type of oppression that members of multiply-oppressed groups (e.g., Black women) face operates in ways that are not simply summations of the types of oppression faced by adjacent singly-oppressed groups (e.g., White women and Black men).

The conceptual backbone for both PHCRP and DJ is critical race theory (CRT), which conceptualizes American society as intrinsically racist, rather than unnaturally distorted by racism from some underlying neutral state.^13^ CRT posits that the dominant social, economic, and political structures in contemporary society are shaped by racism. The core tenets of CRT are shown at the base of Figure 1, which depicts how PHCRP and DJ draw from CRT foundations to advance equity in public health policies, programs, and practice. The complementarity of PHCRP and DJ is illustrated in the case study that follows.

PHCRP is a semistructured process with four foci (Fig. 1) that can be used independently or in conjunction with other frameworks to maintain a systematic focus on racial justice in public health research.^14^ Although PHCRP was developed to embed antiracism in research, the process is equally applicable to the implementation of policies and programs. To bring about fundamental change, the ownership of program or policy implementation must be shifted away from public health institutions toward communities. The 10 principles of the DJ framework (Fig. 1) provide practical guidance on doing just this.^15,16^

These principles have been advanced by members of the design community in multiple fields who have recognized that solutions using traditional design principles exclude those who are intersectionally disadvantaged. DJ asserts that community members, as recipients, users, or sometimes victims of a product, policy, or program, are best suited to lead design and action to address inequity.^17,18^ A sequential traversal through the four focus areas of PHCRP, mapped onto the different DJ principles, is provided in the first two columns of Table 1.^19^ The third column demonstrates the joint use of these approaches to the case study presented.

**Table 1.** Applying the Design Justice Principles to Coronavirus Disease 2019 Vaccine Distribution

PHCRP	DJ	Application to COVID-19 vaccine distribution
Focus 1: Contemporary patterns of racialization	DJ2: We center the voices of those who are directly impacted by the outcomes of the design process.	Ensuring outreach activities, developed and led by members of the community (e.g., input from Equity and Justice Community Coalition), not only encourage people to get vaccinated but also acknowledge systems of oppression that influence access. This includes educating the community on why those most directly affected by those systems deserve to be first in line, not just for vaccines but also for other services promoting health and well-being.
	DJ3: We prioritize design's impact on the community over the intentions of the designer.	Leveraging processes to collect data on programmatic impact, with an explicit effort to capture the experiences of and impacts on oppressed groups, and ensuring that communication about the vaccine rollout not only includes the numbers of people vaccinated (the intention of state and local authorities) but also tells the stories of those for whom access was difficult and why.
Focus 2: Knowledge production	DJ6: We believe that everyone is an expert based on their own lived experience, and that we all have unique and brilliant contributions to bring to a design process.	Developing multiple events and outlets for community members to submit ideas, experiences, and other information that shape program design (both before program start and to inform ongoing program refinement), and ensuring these outlets explicitly accommodate community availability, work hours, technology access, and language needs.
	DJ4: We view change as emergent from an accountable, accessible, and collaborative process, rather than as a point at the end of a process.	Explicitly designing collaboration processes (e.g., Equity and Justice Community Coalition) as a structure for ongoing communication and accountability around equity issues beyond vaccine distribution or the pandemic.
	DJ5: We see the role of the designer as a facilitator rather than an expert.	Using design expertise to focus and strengthen the representation of ideas and inputs by community members, not to solve problems for the community, and giving community members explicit opportunities to lead program design and implementation.
	DJ10: Before seeking new design solutions, we look for what is already working at the community level.	Avoiding the creation of new structures unless absolutely necessary, and ensuring the design solution complements and leverages existing networks and programs that are already working to challenge power hierarchies within the community.
Focus 3: Conceptualization and measurement	DJ7: We share design knowledge and tools with the communities.	Building local leadership and expertise by engaging community members as co-facilitators when using design knowledge and tools to advance future capacity to address health inequity and racial justice beyond vaccine distribution.
Focus 4: Action	DJ1: We use design to sustain, heal, and empower our communities, as well as to seek liberation from exploitative and oppressive systems.	Developing action plans for long-term use of the solution structure and processes (e.g., the Equity and Justice Community Coalition), including processes on community outreach, convening, and information gathering, to ensure addressing inequity and racism beyond vaccine distribution is seen as a critical priority.
	DJ8: We work toward sustainable, community-led, and -controlled outcomes.	Using the vaccine distribution effort to create paid and volunteer opportunities for community members with the goal of building broad and sustainable capacity for equity-focused systems change. Ensuring communities have sustained, dedicated (beyond just grant funding) financial, human, and technical resources needed to carry out community-led work.
	DJ9: We work toward nonexploitative solutions that reconnect us to the earth and to each other.	Using the experiences from the outreach, communication, oversight, and advocacy activities undertaken during the vaccine distribution process to identify and strengthen community networks committed to challenging and disrupting exploitative power structures.

COVID-19, coronavirus disease 2019; DJ, design justice; PHCRP, public health critical race praxis.

Importantly, transferring leadership from those traditionally seen as experts to community members does not absolve public health practitioners from playing a critical role in advancing equity. As public health practitioners we must actively engage as coproducers of antiracist solutions by examining our positionality (i.e., social position and power) within institutions, communities, and systems; disrupting our own spheres of power and privilege; and ensuring that communities have the resources they need not only to lead but also to implement and sustain the solutions they create.

## Applying DJ Principles to COVID-19 Vaccination: A Thought Experiment

In grappling with stark vaccine inequity, U.S. states and local communities have begun to develop and implement strategies aimed at driving equitable vaccine distribution and administration. Although some of these efforts have fallen flat—such as the example presented in the opening of this piece—others have seen success.^20^ In the following section, we demonstrate the ideas presented so far through an example involving community-led COVID-19 vaccine distribution. This example is intended to illustrate how any public health intervention can be used as a stimulus for disrupting structural patterns of inequity.

Consider a county that receives COVID-19 vaccines and distributes them to community outlets for administration. Media attention to inequitable vaccine access among Black, Indigenous, and People of Color (BIPOC) communities leads the county to take a proactive stance. The county engages BIPOC leaders and community organizations around an equity strategy to address vaccine hesitancy and improve vaccine access. However, despite its apparent attractiveness, the county's approach does not result in the vaccines reaching the intended populations.

Why might this be the case? With the matrix of domination structuring access to health care, efforts that fail to recognize and disrupt power cannot remedy disparities—even if community leaders employ a variety of mechanisms to make vaccines accessible, such as online schedulers, vaccine appointment hotlines, or walk-in pop-up clinics. Each can be overrun and usurped by better-resourced individuals from outside the community.^21,22^ Indeed, those sitting in positions of advantage due to a complex combination of race, profession, income, and geography can maneuver preferential access to a highly sought-after life-saving commodity, even when it is earmarked for those who are persistently disadvantaged.

Rather than laying out an “equitable” solution that produces inequitable results, a DJ-based solution operating within the principles of PHCRP would intentionally map out structural patterns of oppression and explicitly design the vaccine distribution process to challenge these patterns.

For example, the county might first build an Equity and Justice Community Coalition to provide oversight and leadership. This Coalition would include BIPOC business owners and residents with the greatest need—using what Wolfe and colleagues define as a “community development approach in which residents have equal power in determining the Coalition's agenda and resource allocation.”^23^ The work of the Coalition would be facilitated by public health practitioners with experience using design tools for social change, such as power mapping, resource flow or assets mapping, and co-creation sessions.^24,25^

Table 1 outlines the actions that the Coalition might take to make equity integral to vaccine distribution. Applying DJ principles 2 and 3, the Coalition would use data and stories about patterns of oppression to identify and prioritize those who should be first in line for the vaccine. In accordance with DJ principles 4, 5, 6, and 10, the Coalition would favor extensive use of existing local networks and social relationships for outreach and continual community input. DJ principle 7 encourages community leader skill development to drive equity-focused change in other areas in the community. This will require a proactive effort to select and engage community members to be coproducers and coleaders from the beginning. Following these principles with intention and purpose will achieve the ultimate goals laid out in DJ principles 1, 8, and 9, which is to strengthen and resource community capacity for sustained action to disrupt structural racism and intersectional domination to ignite real social change.

## Recommendations for the Path Ahead: Envisioning a Different Future

Public health's efforts to create outcomes that diminish health disparities will fall flat if they ignore the underlying power differentials that produce inequity. To be a force for change, public health practitioners must commit to explicitly addressing systemic and institutional racism and other inequitable power dynamics. Although the examples we have provided are domestic, power and inequity are global phenomena and thus have global implications.

With the concepts and tools presented in this article—CRT as a conceptual frame, PHCRP as a systematic approach to adapting CRT for public health applications, and DJ to ensure the leadership from those who suffer the most—public health can be a force for justice. But change cannot take place unless practitioners intentionally engage in a role shift, from passive process enablers to active coconspirators in dismantling unjust power structures. The time is ripe for public health to take up this charge.

## Abbreviations Used

BIPOC: Black, Indigenous, and People of Color
COVID-19: coronavirus disease 2019
CRT: critical race theory
DJ: design justice
PHCRP: public health critical race praxis
